# Chelerythrine Chloride: A Potential Rumen Microbial Urease Inhibitor Screened by Targeting UreG

**DOI:** 10.3390/ijms22158212

**Published:** 2021-07-30

**Authors:** Xiaoyin Zhang, Yue He, Zhanbo Xiong, Min Li, Ming Li, Nan Zheng, Shengguo Zhao, Jiaqi Wang

**Affiliations:** State Key Laboratory of Animal Nutrition, Institute of Animal Sciences, Chinese Academy of Agricultural Sciences, Beijing 100193, China; zhangxiaoyin1210@webmail.hzau.edu.cn (X.Z.); 82101182379@caas.cn (Y.H.); 82101201506@caas.cn (Z.X.); limin@webmail.hzau.edu.cn (M.L.); liming01@caas.cn (M.L.); zhengnan@caas.cn (N.Z.)

**Keywords:** urease inhibitor, UreG, chelerythrine chloride, ammonia production, rumen

## Abstract

Inhibition of ruminal microbial urease is of particular interest due to its crucial role in regulating urea-N utilization efficiency and nitrogen pollution in the livestock industry. Acetohydroxamic acid (AHA) is currently the only commercially available urease inhibitor, but it has adverse side effects. The urease accessory protein UreG, which facilitates the functional incorporation of the urease nickel metallocentre, has been proposed in developing urease inhibitor through disrupting urease maturation. The objective of this study was to screen natural compounds as potential urease inhibitors by targeting UreG in a predominant ruminal microbial urease. In silico screening and in vitro tests for potential inhibitors were performed using molecular docking and an assay for the GTPase activity of UreG. Chelerythrine chloride was selected as a potential urease inhibitor of UreG with an inhibition concentration IC_50_ value of 18.13 μM. It exhibited mixed inhibition, with the K_i_ value being 26.28 μM. We further explored its inhibition mechanism using isothermal titration calorimetry (ITC) and circular dichroism (CD) spectroscopy, and we found that chelerythrine chloride inhibited the binding of nickel to UreG and induced changes in the secondary structure, especially the α-helix and β-sheet of UreG. Chelerythrine chloride formed a pi-anion interaction with the Asp41 residue of UreG, which is an important residue in initiating the conformational changes of UreG. In conclusion, chelerythrine chloride exhibited a potential inhibitory effect on urease, which provided new evidence for strategies to develop novel urease inhibitors targeting UreG to reduce nitrogen excretion from ruminants.

## 1. Introduction

Urea is commonly used as a cost-efficient replacement of feed proteins to provide the sole nitrogen source for urease [1,2,3]. In rumens, urease catalyzes the hydrolysis of urea into ammonia, which can be synthesized into microbial protein to support the requirements for animal growth, meat or milk production [4]. However, the high urease activity leads to excessive ammonia production, which not only results in the explosion of toxic ammonia in the blood, but is also converted to volatile ammonia and escapes into the environment [5,6]. Emissions of nitrogen have been recognized as one of the major drivers in the production of greenhouse gases and wastewater and soil pollution. The livestock industry contributes approximately 65 Tg·N·yr^−1^, which is roughly one-third of the global human-induced nitrogen emissions [7]. Ruminant nitrogen is the major source of livestock pollution, with approximately 46 Tg·N·yr^−1^, equivalent to 71% of the total nitrogen emissions from livestock [7]. For ruminants, the dietary nitrogen utilization efficiency is only about 25% [8,9], with as much as 60–90% of the feed nitrogen being excreted [10]. Taken together, regulation of urease activity is crucial to reduce ammonia emissions and improve the efficiency of urea-N utilization in ruminants, and urease inhibitors have been recognized as one of the most effective strategies.

There are many kinds of urease inhibitors (Table S1), including those that attack the active center of urease, such as urea analogues [11], hydroxamic acids [12], phosphoramide and their derivatives [13], as well as other inhibitors that interact with the flap regions near active centers, such as 1, 4-benzoquinone [14], catechol [15], and some heavy metal ions [16]. However, only acetohydroxamic acid (AHA) is a commercially available urease inhibitor approved by the Food and Drug Administration for the treatment of humans and animals [17]. Unfortunately, significant adverse effects, including deep-vein phlebothrombosis, lower-extremity phlebitis, and malformation of embryos, have been reported [18]. In rumens, AHA was found to degrade rapidly, and inhibit the growth of rumen microorganisms other than urease-producing bacteria [19]. In recent years, plant-derived natural compounds (including that shown in Graphical Abstract [20,21,22,23]) have been preferred for their low toxicity, chemically stability, eco-friendliness and high efficiency at low concentrations [24]. Gnetegha Ayemele et al. [25] screened giant milkweed as an alternative plant-derived feed additive, which could improve nitrogen utilization efficiency by inhibiting protozoa in ruminants without impairing fermentation. Limited studies have found that natural compounds such as terpenoids, phenolic compounds, alkaloids, and other substances were inhibitory towards the activities of plant and microbial ureases [26]. These findings suggest that natural compounds have great potential as urease inhibitors.

The active center of urease is deeply buried in apo-urease, with an unobserved wide-open state [27]. Additionally, urease is a highly specific substrate of urea [28], which makes it challenging to design new urease inhibitors based on the active center. Accessory protein UreG, which is a GTPase involved in transferring nickel to the active center and activating urease upon GTP hydrolysis, plays an important role in urease maturation [29]. It has been reported [30,31] that UreG receives nickel from the accessory protein UreE through the formation of an UreE-UreG complex. Subsequently, nickel-charged UreG delivered nickel to the active center of apo-urease by forming an UreG-UreFH complex and apo-urease/UreGFH super-complex, in which GTP hydrolysis induced the conformational changes of UreG, and promoted the insertion of nickel into the active center. Moreover, UreG and urease maturation are relatively conserved among different urease-producing bacteria [32]. Disruption of the urease maturation process by abolishing UreG function has been proposed as a new strategy to develop urease inhibitors.

We identified a predominant urease gene cluster from an uncultured ruminal bacterium using metagenome, and revealed the characteristics of UreG interacting with UreE as well as transferring nickel previously [33]. This study aimed to select potential ruminal microbial urease inhibitors from natural compounds by using UreG as a regulatory target. A natural compound was identified by detecting GTPase activity of UreG and virtual screening using molecular docking. The inhibition mechanisms were evaluated by kinetic study. Whether this compound could affect nickel binding to UreG, as well as the influencing mechanism were further explored. This study provides a new strategy of screening ruminal microbial urease inhibitors, and a novel natural compound of regulating ruminal urease activity.

## 2. Results

### 2.1. Screening of Urease Inhibitor

**Figure 1:**
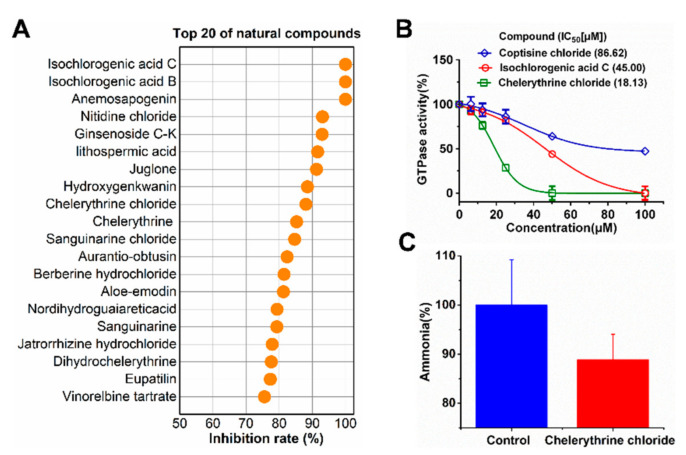
Effect of natural compounds on GTPase activity of UreG and ammonia emission. (**A**) Inhibition rates of the top 20 natural compounds on UreG GTPase activity. In this process, 100 μM natural compound was added to GTPase assay buffer (2 mM MgSO_4_, 300 μM GTP, 200 mM NaCl, 1 mM Tris(2-carboxyethyl)phosphine (TCEP), 10 mM KHCO_3_, 20 μM NiSO_4_, 20 mM HEPES, pH 7.5) containing 3 μM UreG. (**B**) Inhibition of GTPase activity of UreG by isochlorogenic acid C and chelerythrine chloride. Coptisine chloride is shown for comparison. The IC_50_ values against GTPase activity were calculated at different concentrations of natural compounds (100, 50, 25, 12.5, 6.25, and 0 μM). Results are desired as means ± SD of triplicate tests. (**C**) Effect of chelerythrine chloride on the ammonia release of ruminal microbial crude protein. The final concentrations of ruminal microbial crude protein and chelerythrine chloride were 176 and 7.8125 μM, respectively.

A set of 1130 natural compounds were collected for preliminary screening of UreG inhibition by detecting their inhibition rate. Given that 1130 natural compounds would consume a large amount of UreG, these screening tests were not repeated. Then, the top 48 compounds with a strong inhibitory rate were re-screened in triplicate. The top 20 natural compounds are shown in Figure 1A. Isochlorogenic acid C, isochlorogenic acid B, and anemosapogenin had a higher inhibitory effect than others, and inhibited the GTPase activity of UreG by almost 100%. Considering the cost performance, application prospects, sources and categories of the compounds, isochlorogenic acid C and chelerythrine chloride were selected for further analysis.

A previous experiment [34] demonstrated that coptisine could inhibit the function of UreG during urease maturation; thus, GTPase activity of UreG with coptisine chloride was used as a positive control during the measurement of IC_50_. As shown in Figure 1B, isochlorogenic acid C and chelerythrine chloride exhibited greater inhibitory potency with IC_50_ values lower than that of the positive control. Chelerythrine chloride was the best inhibitor, with an IC_50_ value of 18.13 μM, showing 4.78-fold more potency than coptisine chloride. Interestingly, although the inhibitory potency of isochlorogenic acid C was similar to that of chelerythrine chloride at 100 μM, chelerythrine chloride exerted better inhibition at lower concentrations.

To gain insight into the potential of the identified compounds for further developing as UreG inhibitors, the three compounds (coptisine chloride, isochlorogenic acid C, and chelerythrine chloride) were selected to test the potential binding affinity and inhibitor constant (K_i_) against UreG using molecular docking, and the results are shown in Table 1. The highest negative value of binding energy was considered as the interaction of ligand and receptor with maximum binding affinity. The most potent compound, chelerythrine chloride, found in the UreG inhibition assay, not only performed the strongest binding affinity with UreG, but also showed the lowest K_i_ value. Furthermore, chelerythrine chloride inhibited the ammonia release of ruminal microbial crude protein (Figure 1C). These data indicated that chelerythrine chloride had a greatest potential to be used as an UreG inhibitor.

### 2.2. Kinetic Study of UreG Inhibition by Chelerythrine Chloride

**Figure 2:**
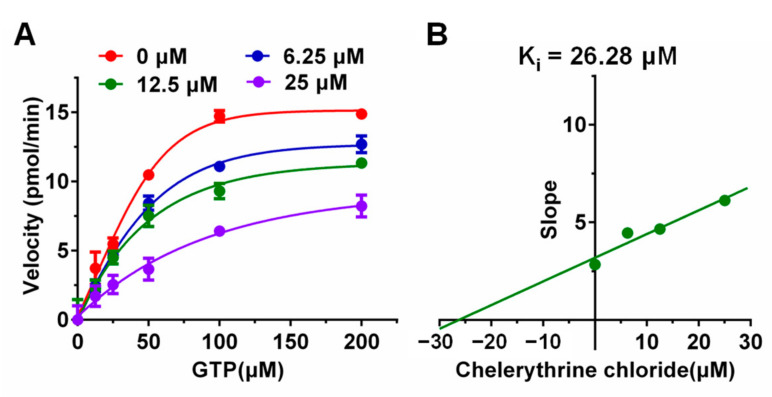
Kinetic study of UreG inhibition by chelerythrine chloride. (**A**) The rates of GTP hydrolysis were plotted as a function of GTP concentrations ranging from 0 to 200 μM, at different concentrations of chelerythrine chloride (0, 6.25, 12.5, and 25 μM). Results are desired as means ± SD of triplicate tests. (**B**) The inhibition constant K_i_ was calculated as the intersection on x-axis of the plot of 1/V_max_ versus chelerythrine chloride.

The inhibitory effect of chelerythrine chloride derivatives on the GTPase activity of UreG was further examined to investigate the inhibitory potential, kinetics studies and inhibitory mechanisms in GTPase assay buffer. As shown in Figure 2A, the UreG activity increased with the increase in substrate GTP, and chelerythrine chloride inhibited the UreG activity in a concentration-dependent manner. The K_m_ and V_max_ values from the Lineweaver–Burk plots are summarized in Table S2, and the data revealed that chelerythrine chloride was a mixed-type inhibitor for UreG, in which K_m_ and V_max_ decreased gradually after adding 6.25, 12.5 and 25 μM of chelerythrine chloride, while both K_m_ and V_max_ values without a compound were smaller than those with 6.25 μM chelerythrine chloride. Furthermore, the inhibition constant K_i_ value was calculated using the slopes of each Lineweaver–Burk line, and the obtained K_i_ value was 26.28 μM for chelerythrine chloride (Figure 2B).

### 2.3. Chelerythrine Chloride Inhibited Nickel Binding to UreG

UreG is thought to act as a bridge that transfers nickel from UreE to apo-urease upon the formation of the UreE-UreG and UreDFG complexes, and nickel binding to UreG plays a crucial role in urease maturation. We therefore investigated whether chelerythrine chloride could affect the interaction between UreG and nickel. An isothermal titration calorimetry (ITC) experiment was performed, in which nickel was titrated into UreG in the presence of chelerythrine chloride, and titration of UreG with nickel was set as the control group.

**Figure 3:**
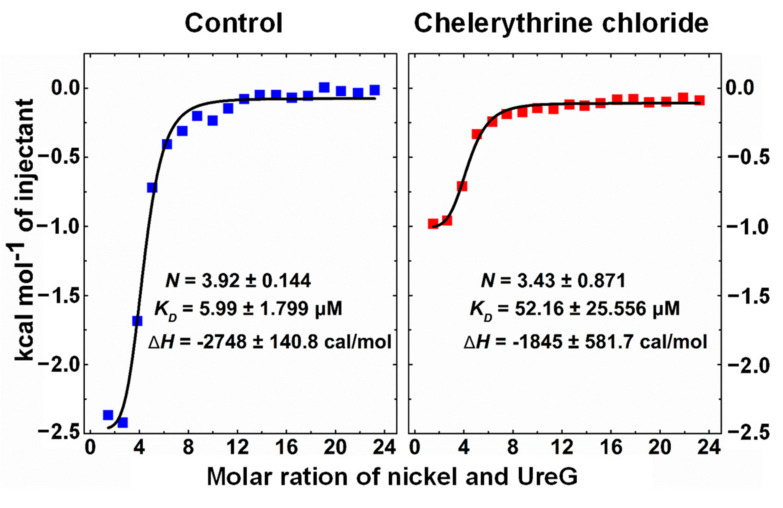
Isothermal titration calorimetry (ITC) enthalpograms of nickel binding to UreG with (right) or without (left) chelerythrine chloride. Titration data are presented as colored squares and fits as black solid lines. The molar ratio (*N*), equilibrium dissociation constant (*K_D_*) and enthalpy change (Δ*H*) were shown in the inset.

The heat change data were fitted with a one-site binding model, fitted curves were generated, and the thermodynamic parameters are shown in Figure 3. Chelerythrine chloride did not significantly alter the molar ration (*N*) of nickel binding to UreG, while the binding affinity was 8.7-fold weaker compared to the control group. In addition, the vertical axis represents the heat change for each drop of nickel that is injected into the sample cell, and the heat released for UreG with chelerythrine chloride was lower than that of UreG without the inhibitor; a similar result was found for the −Δ*H* value. These observations suggest that chelerythrine chloride inhibited nickel binding to UreG, meaning that the urease maturation process would be disrupted by chelerythrine chloride through blocking nickel transfer.

### 2.4. Chelerythrine Chloride-Induced Secondary Structure Change of UreG

**Figure 4:**
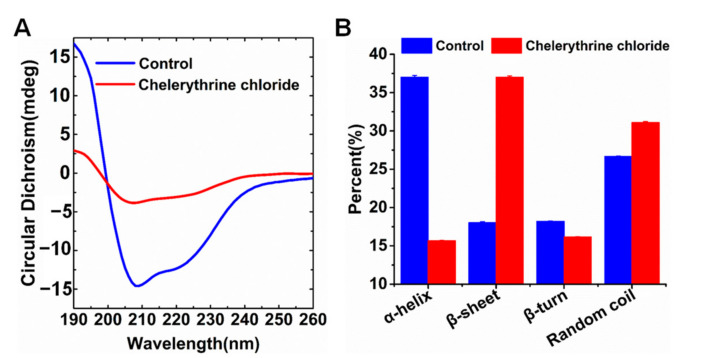
Effect of chelerythrine chloride on second structure of UreG. (**A**) Circular dichroism (CD) spectroscopies of UreG with (red) or without (blue) chelerythrine chloride. (**B**) The composition of the secondary structure in UreG with (red) or without (blue) chelerythrine chloride.

To explore the possible mechanisms by which chelerythrine chloride prevented nickel binding to UreG, changes in the secondary structure of UreG were first detected using circular dichroism (CD) spectroscopy. The CD spectra of UreG in the absence (blue curve) and the presence of chelerythrine chloride (red curve) are shown in Figure 4A. The UreG curve showed two large negative peaks at 208 and 222 nm, which is a clear signature of the existence of α-helix present in the protein. The addition of chelerythrine chloride caused a great change in the intensity of the CD signal, accompanied by a slight left shift at 208 nm. The composition of the secondary structure in the protein is shown in Figure 4B; the UreG consisted of ~37.02% α-helix, ~18.05% β-sheet, ~18.21% β-turn, and ~26.69% random coil. There was a ~21.36% decrease in the α-helical content and a ~18.97% increase in the β-sheet content upon the addition of chelerythrine chloride, and the variations in the β-turn and random coil were slight. In general, the addition of chelerythrine chloride changed the overall CD spectrum of UreG, especially in the α-helix and β-sheet.

### 2.5. Molecular Docking of Chelerythrine Chloride towards UreG

**Figure 5:**
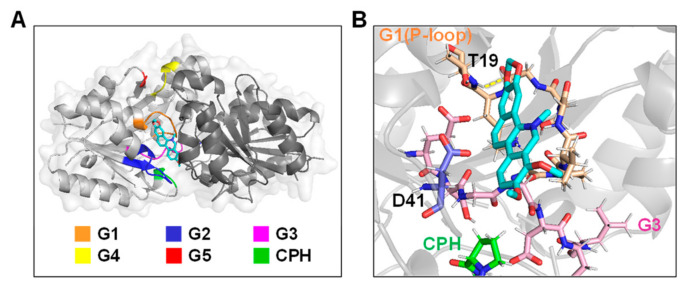
Binding mode of chelerythrine chloride with UreG. (**A**) Location diagram of chelerythrine chloride in UreG dimer. (**B**) Interactions between chelerythrine chloride and UreG. The structure of chelerythrine chloride is colored in cyan. Residue Thy19 makes a strong hydrogen bonding interaction (yellow dotted line) with an O atom of chelerythrine chloride. Conserved motifs of G1-G5 and CPH metal binding motif are colored as indicated.

We next investigated the potential binding interfaces between chelerythrine chloride and UreG using molecular docking. The best possible binding modes of chelerythrine chloride towards UreG were shown as both enzyme surface (Figure 5A) and cartoon mode (Figure 5B), respectively. Chelerythrine chloride was located near the G1 motif, G2 motif and G3 motif, in which the main chains of the G1 motif and G3 motif wrapped around chelerythrine chloride. An O atom of chelerythrine chloride was found to make a strong hydrogen bonding interaction with Thy19 in the G1 motif, with a distance of 2.2 Å. The side chain of Asp41 in the G2 motif formed a pi-anion interaction with chelerythrine chloride, and the distance was between 2.8 and 4.5 Å. Interestingly, we previously found [33] that the amino acid residue Asp41 not only abolished the GTPase activity of UreG, but also affected nickel binding to UreG. These results suggest that chelerythrine chloride inhibited nickel binding to UreG by interacting with Asp41.

## 3. Discussion

In recent years, the safety of food, medical treatment, chemical industry, and agriculture has become a concern for consumers. Plant-derived natural compounds and their secondary metabolites have received considerable attention because of their natural properties, and have been intensively investigated and widely used in anti-inflammatory [35], anti-cancer [36], antiviral [37] and other biomedical fields. With the advent of the non-antibiotic era, the supervision of feed additives is becoming stricter in the livestock industry. Some natural compounds, including essential oils, tannins, and saponins, have been reported as non-antibiotic feed additives to improve rumen fermentation, mitigate methane excretion and nitrogen emission, and enhance feed utilization efficiency [38,39,40], which provides a new direction for the design and synthesis of rumen microbial urease inhibitors.

Apo-urease is composed of an α subunit, β subunit, and γ subunit, with the active center being located in the α subunit. The crystal structures of apo-urease showed a typical quaternary structure formed through a minimal trimeric configuration, such as the ((αβ)_3_)_4_ structure of *H. pylori*, and the (αβγ)_3_ structure of *K. aerogenes* [41]. The active center is deeply buried in the supramolecular assembly of urease, and has an unobserved wide-open state, which makes it very difficult to attack with urease inhibitors. The only commercially available inhibitor, AHA, requires a high dose to treat urinary tract infections, with a relatively low inhibitory effect [21], which is attributable to the difficulty of coming into contact with the deeply buried active center. Apo-urease is inactive; its activation involves nickel delivery to the active center and requires the cooperation of at least four urease accessory proteins: UreD (or UreH in *Helicobacter* sp.) UreE, UreG and UreF. These accessory proteins are generally small proteins, which might serve as greater targets for developing urease inhibitors than apo-urease itself. Wang [42] reported that inhibiting human copper trafficking proteins with a small molecule significantly attenuates cancer cell proliferation, indicating that copper chaperones can be a target for anticancer therapies.

The accessory protein UreG is a GTPase, and its GTPase activity is highly consistent with its biological role in urease activation, which involves nickel transfer among accessory proteins, insertion of nickel into the active site, and conformational changes depending on GTP hydrolysis [30,31]. Given the importance of UreG GTPase activity and the simplicity of GTPase activity detection, we set the GTPase activity of UreG as an indicator to preliminary screen for potential inhibitors from 1130 natural compounds. Most of the top 20 compounds with better inhibition potency were alkaloids, among which nitidine chloride, chelerythrine chloride, chelerythrine, sanguinarine chloride, sanguinarine and dihydrochelerythrine were the major bioactive ingredients of *Zanthoxylum nitidum*. Some studies [43,44] found that *Z. nitidum* and its bioactive ingredients were used to treat gastritis diseases, and inhibited the growth of *H. pylori*. The mechanism of anti-gastritis and inhibiting *H. pylori* activity remains unclear, which may be related to the decrease in UreG GTP activity.

To better identify potent inhibitors of urease, the IC_50_ of compounds with stronger inhibition potency towards UreG GTP activity, as well as the binding energy and inhibitor constant obtained using molecular docking, were used for further screening. Chelerythrine chloride is considered to be the most potential inhibitor of urease, with the lowest IC_50_ and K_i_ values and the highest binding energy. Li et al. [34] demonstrated that when the final concentration of *H. pylori* UreG was 5 μM, the IC_50_ value of coptisine was 89.86 μM. In this study, the IC_50_ value of coptisine chloride was 86.62 μM, with a final concentration of 3 μM UreG, and the IC_50_ value of 18.13 μM for chelerythrine chloride was significantly lower than that of coptisine chloride. Additionally, chelerythrine chloride and coptisine chloride, two alkaloids, had similar binding energy and K_i_, which were different from that of the caffeoylquinic acid isochlorogenic acid C. Notably, when the final concentration of natural compounds was 100 μM, the inhibition rate of isochlorogenic acid C against UreG activity was stronger than that of chelerythrine chloride (Figure 1A). However, when their final concentration was lower than 100 μM, the fitting curve of chelerythrine chloride was steeper with weaker GTPase activity (Figure 1B), suggesting that it is better to evaluate the inhibition potency of compounds using multiple additive concentrations.

UreG is thought to act as a bridge that transfers nickel from UreE to apo-urease upon the formation of the UreE-UreG and UreDFG complexes. The key to UreG serving as a target to design urease inhibitors is to disrupt the urease activation process by blocking nickel delivery to UreG. Here, we further explored whether nickel binding to UreG was suppressed by chelerythrine chloride as well as its underlying mechanism. The ITC results show that chelerythrine chloride reduced the molar ratio, binding affinity and −Δ*H* value in between nickel and UreG, inhibiting nickel binding to UreG. Furthermore, chelerythrine chloride induced a secondary structure change of UreG, and formed a pi-anion interaction with Asp41. The crystal structure of the UreGFH complex [31] revealed that the Asp37 residue (Asp41 residue in the study) generated charge-charge repulsion with γ-phosphate upon GTP binding, which induced the formation of a salt bridge between Glu42 and Arg130, and the motion of “zip-up” between β2 strand and β3 strand, subsequently propagating the conformational changes of UreG in the CPH motif, which was the key site for nickel binding. Chelerythrine chloride may disrupt the biological function of CPH motif through the induced structure change of UreG and interaction with Asp41 residue. Our previous study [33] also found that the Asp41 residue was the key residue affecting the binding of nickel and UreG. A recent study [45] demonstrated that the cmpd4 compound formed a hydrogen bond with Thy15 residue of *H. pylori* UreG, and inhibited the activities of UreG and *H. pylori.* The results of Thy15 are consistent with those of our Thy19. 

Yang et al. [45] first proposed and confirmed that targeting the metallochaperone UreG to design urease inhibitors could disrupt the urease maturation process, and provided two effective urease inhibitors of colloidal bismuth against *H. pylori* activity only through UreG, not apo-urease. Li et al. [34] demonstrated that coptisine inactivation of *H. pylori* urease involved binding to the urease active site sulfhydryl group and accessory protein UreG. These results show the potential of UreG serving as a target for developing urease inhibitors. Chelerythrine chloride not only inhibited the GTPase activity of UreG from a predominant ruminal microbial urease, but also suppressed nickel binding to UreG, which would interfere with urease maturation. Moreover, in clinical practice, the antimicrobial, antibacterial, anti-inflammatory and antiplatelet activities of chelerythrine have been extensively studied [43,46]. In the livestock industry, pharmacokinetic analysis concluded that the addition of chelerythrine to feed was safe due to the first pass effect after intestinal and liver metabolism [47]. These characteristics of chelerythrine chloride provide a solid foundation for the development of chelerythrine chloride as a natural feed additive in ruminants.

## 4. Materials and Methods

### 4.1. Preparation of Urease Accessory Protein UreG

The accessory protein UreG (GenBank ID: MN660252) was identified, overexpressed, and purified as described previously [33]. UreG was cloned into the pASK-IBA5C plasmid (IBA, Goettingen, Germany) and expressed as a Strep-tagged protein using transformed *Escherichia coli* BL21 (DE3) cells (Weidi Biotechnology, Shanghai, China). The expression plasmid containing Strep-UreG was induced by anhydrotetracycline (IBA, Goettingen, Germany) and lysed via sonication in binding buffer (10 mM Tris-HCL, 150 mM NaCl, 1 mM EDTA, pH 8.0). Crude cell lysates were further purified using Strep-Tactin beads according to the manufacturer’s instructions (Beaver, Suzhou, China). The final eluted proteins were incubated in 20 mM ethylene diamine tetraacetic acid (EDTA) and 1 mM DL-dithiothreitol (DTT) overnight at 4 °C to obtain the apo-form of the proteins, which were collected and buffer-exchanged into HBS buffer (GE, Boston, MA, USA) using a 3 kDa centrifugal filter device (Millipore, Billerica, MA, USA). 

### 4.2. GTPase Activity of UreG

GTPase activity of UreG was determined using a Malachite Green Phosphate Assay Kit (Sigma-Aldrich, St. louis, MI, USA) under various conditions. In this process, 3 μM of UreG was incubated with the same amount of different natural compounds in GTPase assay buffer (2 mM MgSO_4_, 300 μM GTP, 200 mM NaCl, 1 mM TCEP, 10 mM KHCO_3_, 20 μM NiSO_4_, 20 mM HEPES, pH 7.5) for 30 min at room temperature, then the free phosphate from hydrolysis of GTP was determined by measuring the absorbance at 620 nm. To eliminate the potential effect of natural compounds and GTPase assay buffer on the malachite green assay, the measured result included a subtraction of absorbance of reaction with inactivated UreG (after boiling) incubated the same compounds. The inhibition rate was determined by using the following formula:

$$\mathrm{Inhibition}\;\mathrm{rate}\;(\%)=\left(1-\frac{A-C}{B-C}\right)\times 100$$

where *A* represents the absorbance of the reaction with the natural compound, *B* represents the absorbance of the reaction without the natural compound, and *C* is the absorbance of the corresponding blank control (inactivated UreG). Each sample was assayed three times, with each measurement performed in triplicate.

To screen the potential inhibitor from the natural compounds, further measurements were carried out to obtain the half inhibition concentration of compounds (IC_50_). The IC_50_ values against GTPase activity were calculated in the presence of different concentrations of the natural compounds (100, 50, 25, 12.5, 6.25, and 0 μM) using GraphPad Prism (v. 8.0.1). For comparison, the GTPase activity of UreG without the natural compound was set at 100%.

### 4.3. Preparation of Ruminal Microbial Crude Protein and Measurement of Ammonia Released

Ruminal digesta samples were collected as described previously [48]. A 10 mL aliquot for the sample was used for protein extraction by ultrasonication (6 s each with 6 s intervals using 100 W on ice, total 10 min). The supernatant was collected by centrifugation at 16,000× *g* at 4 °C for 10 min, which was called ruminal microbial crude protein. The concentration of ammonia released was measured by a modified phenol/hypochlorite reaction method [49]. The ammonia release of rumen microbial protein without the chelerythrine chloride was set at 100%.

### 4.4. Kinetic Study

The kinetic mechanism of UreG inhibition by chelerythrine chloride was determined using the kinetic assay method. The concentrations of chelerythrine chloride used were 25, 12.5, 6.25, and 0 μM (covering the IC_50_ value), while the concentrations of the substrate (GTP) were 200, 100, 50, 25, 12.5, and 0 μM. The values of the kinetic parameters (K_m_ and V_max_) were calculated from the Lineweaver–Burk plots by using GraphPad Prism, version 8.0.1 (GraphPad Software Inc., San Diego, CA, USA), and the inhibition constant (K_i_) was determined as the intersection on the x-axis of the plot of 1/V_max_ versus different concentrations of chelerythrine chloride. The variations of K_m_ and V_max_ were used to determine the type of enzyme inhibition. If the K_m_ values increase as the inhibitor concentrations increase, but V_max_ is unaffected, this inhibition type is called competitive inhibition [50,51,52]. If K_m_ is not changed, while the V_max_ values increase with increasing inhibitor concentrations, this is called non-competitive inhibition [53]. The other case, where both K_m_ and V_max_ decrease gradually when adding an increasing concentration of inhibitor, is called uncompetitive inhibition [54]. If the situation is different from the above three cases, the type of inhibition is mixed inhibition.

### 4.5. Molecular Docking

The 3D structure of UreG was modeled using the SWISS-MODEL server, in which *Kp*UreG (PDB ID: 5XKT) was the template, with 77.39% sequence identity. The values of global model quality estimate (GMQE) and qualitative model energy analysis (QMEAN) were 0.92 and −0.15, respectively. The structures of natural compounds were downloaded from the Zink website (http://zinc.docking.org/, accessed on 30 July 2021). AutoDockTools (v.1.5.6) (Scripps, San Diego, CA, USA) was used for molecular docking studies of compounds and UreG, the receptor (UreG) was considered as a rigid structure molecule, and the ligand (natural compound) was considered as a flexible molecule. During docking, UreG receptor was used to delete water molecules, add polar hydrogens, and to calculate the gasteiger charges. All ligands were treated by adding hydrogens, gasteiger charges, and atom types. The application of grid box was to set the docking area of UreG with the search box of size (126, 80, 106) centered at (−1.515, 15.035, −20.547), and the grid point spacing was 0.375 Å. After that, the molecular docking was calculated by the genetic algorithm (GA) method, in which the number of GA runs was 10, the GA population size was 150, and maximum number of energy evolutions was set to 25,000,000. Ten poses were generated for each compound with different binding energy, and the lowest energy structure was considered as the best docking pose [55]. The software AutoDockTools was used to predict the inhibition constant (K_i_) of small compounds and proteins. To better understand the interactions between the natural compound and UreG, the 3D docking results were analyzed using PyMol molecular graphics system.

### 4.6. ITC Measurements

To monitor whether chelerythrine chloride would affect the binding of nickel to UreG, ITC measurements were employed using an AutoITC200 microcalorimeter (GE, Boston, MA, USA). Except for the concentration of NiSO_4_ and UreG, the procedures for nickel binding to UreG were the same as those described previously [33], and 27 μM of UreG was titrated with 1500 μM NiSO_4_ in this study. The effects of chelerythrine chloride on Ni-UreG interaction were examined by adding 238 μM chelerythrine chloride to 27 μM sample of UreG and 1500 μM titrated sample of NiSO_4_, respectively. ITC measurements were performed at 25 °C with the reference cell filled with the same buffer (2 mM MgSO_4_, 200 μM GTP, 200 mM NaCl, 1 mM TCEP, 40 mM HEPES, pH 7.5) as that used for the experimental samples. The data were fitted with the one-site binding model from the ITC Analysis Module in Origin 7.0 (OriginLab, Hamptons, MA, USA).

### 4.7. CD Spectroscopy

The asymmetrical nature of the peptide bond makes the protein chiral and exhibits the CD phenomenon. CD is frequently used to detect the content of the secondary structure (α-helix, β-sheet, β-turn, random coil) in the protein [56,57,58]. To reduce the potential effect of buffer on CD spectroscopy, the UreG protein and chelerythrine chloride were prepared into distilled water. The final concentrations of UreG and chelerythrine chloride were 233 μg/mL and 88 μM, respectively. CD measurements of UreG with or without chelerythrine chloride were performed using a Chirascan Plus spectrometer (Applied Photophysics Ltd., Surrey, UK) between 190 and 260 nm. The CD spectra were recorded using a 1 mm quartz cell with a data pitch of 1 nm, time-per-point of 0.5 s, and bandwidth of 1 nm. To improve the reproducibility and accuracy, background spectra of the buffer (distilled water with or without chelerythrine chloride) before and after every sample run were recorded and subtracted from the sample spectra. The final data were smoothed using a Savitzky-Golay filter and analyzed using CDNN (v.2.1) software to calculate the percentages of secondary structure in the protein [59].

## 5. Conclusions

This study investigated the inhibition of chelerythrine chloride towards the accessory protein UreG from a predominant ruminal microbial urease. We found that chelerythrine chloride effectively inhibited the GTPase activity of UreG and interaction between UreG and nickel, which were the key steps in disrupting urease maturation. The ammonia release of ruminal microbial crude protein was reduced in the presence of chelerythrine chloride. Further study revealed that chelerythrine chloride was located near the G1, G2, and G3 motif of UreG, forming a pi-anion interaction with Asp41 in the G2 motif, which may interfere with nickel binding to UreG and induce the alteration of the UreG secondary structure. These data suggest that chelerythrine chloride would be a great potential candidate for urease inhibition. In addition, this work was the first to report the inhibition activity of chelerythrine chloride on UreG. To better promote the application of chelerythrine chloride, its associated inhibition mechanisms in various ureases and pharmacokinetics evaluation in different animals need further study.

## Figures and Tables

**Table 1:** Docking parameters of UreG with coptisine chloride, isochlorogenic acid C and chelerythrine chloride.

Compound	CAS Number	Source	Structure	Binding Energy (kcal/mol)	Compound (K_i_)
Coptisine chloride	6020-18-4	Coptidis rhizoma	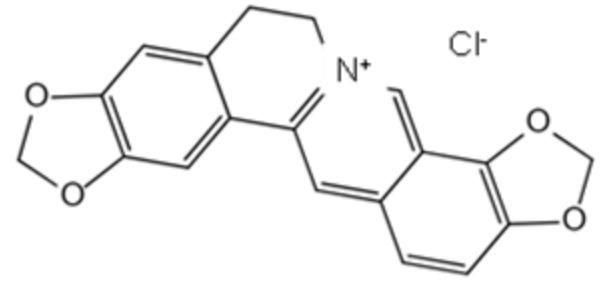	−6.47	17.97 μM
Isochlorogenic acid C	32451-88-0	Honeysuckle	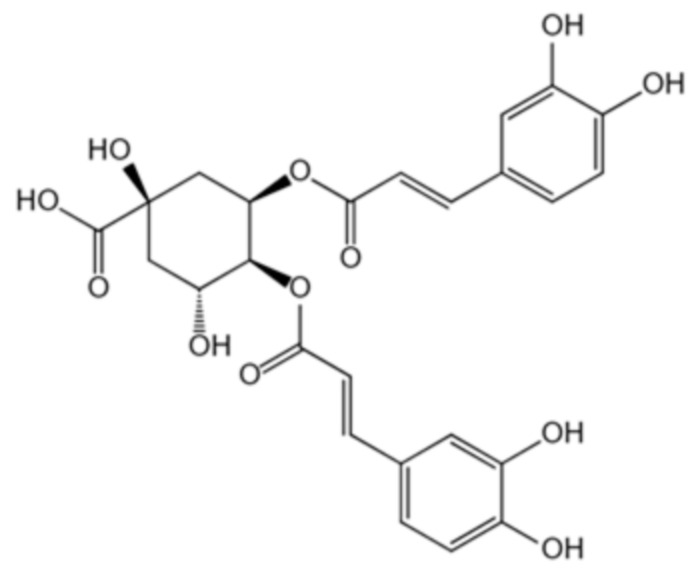	−2.82	8.56 mM
Chelerythrine chloride	3895-92-9	Celandine	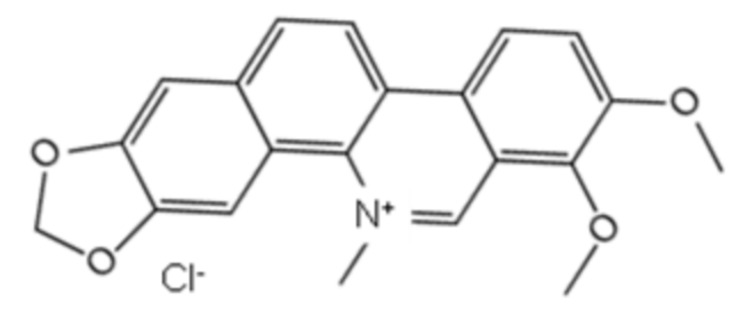	−6.72	11.88 μM

## Data Availability

Not applicable.

## Supplementary Materials

The following are available online at https://www.mdpi.com/article/10.3390/ijms22158212/s1, Table S1: Chemical structures of some urease inhibitors. Table S2: Kinetic parameters of UreG GTPase activity in the presence of different concentrations of chelerythrine chloride (0, 6.25, 12.5, and 25 μM).
