# Supplementary material for: Chelerythrine Chloride: A Potential Rumen Microbial Urease Inhibitor Screened by Targeting UreG
Source: Int J Mol Sci. 2021 Jul 30;22(15):8212. doi: 10.3390/ijms22158212 (PMC8347364; doi:10.3390/ijms22158212)
Supplement: Supplementary file 1 [file ijms-22-08212-s001.zip › ijms-1321142-supplementary.pdf]

**Supplemental material**

Table S1. Chemical structures of some urease inhibitors.

| **Category** | **Urease inhibitor** | **Chemical formula** | **Structure** |
|---|---|---|---|
| urea analogues | Thiourea | $CH_4N_2S$ | 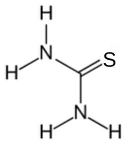 |
| hydroxamic acids | Acetohydroxamic acid （AHA） | $C_2H_5NO_2$ | 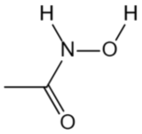 |
| phosphoramide | N-(N-butyl)thiophosphoric Triamide (NBPT) | $C_4H_{14}N_3PS$ | 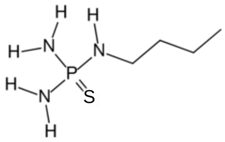 |
| | 1, 4-Benzoquinone | $C_6H_4O_2$ | 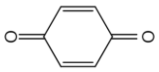 |
| | Catechol | $C_6H_6O_2$ | 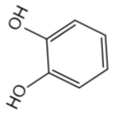 |

Table S2. Kinetic parameters of UreG GTPase activity in the presence of different concentrations of chelerythrine chloride (0, 6.25, 12.5, and 25 μM).

| **Concentration (μM)** | **Equation** | **R square** | $\mathbf{V_{max}}$ | $\mathbf{K_m}$ |
|---|---|---|---|---|
| 25 | y = 6.122x + 0.1158 | 0.9740 | 8.64 | 52.87 |
| 12.5 | y = 4.662x + 0.05278 | 0.9904 | 18.95 | 88.33 |
| 6.25 | y = 4.451x + 0.04340 | 0.9937 | 23.04 | 102.56 |
| 0 | y = 2.836x + 0.04824 | 0.9784 | 20.73 | 58.79 |
